# Adult ADHD with comorbid major depression shows a distinguishable polygenic pattern and negative cognitive style

**DOI:** 10.1038/s41398-026-04008-3

**Published:** 2026-04-04

**Authors:** Thorsten M. Kranz, Rhiannon V. McNeill, Christian P. Jacob, Kira F. Ahrens, Rebecca J. Neumann, Michael M. Plichta, Bianca Kollmann, Fabian Streit, Oliver Tüscher, Klaus Lieb, Heike Weber, Marcel Romanos, Klaus-Peter Lesch, Andreas Reif, Sarah Kittel-Schneider, Georg C. Ziegler

**Affiliations:** 1 Department of Psychiatry, Psychosomatic Medicine and Psychotherapy, University Hospital Frankfurt, Frankfurt am Main, Germany; 2 Department of Psychiatry, Psychosomatics and Psychotherapy, Center of Mental Health, University of Würzburg, Würzburg, Germany; 3 Department of Psychiatry and Psychotherapy, Medius Hospital of Kirchheim, Kirchheim unter Teck, Germany; 4 Department of Psychiatry and Psychotherapy, Johannes Gutenberg University Medical Center, Mainz, Germany; 5 Leibniz Institute for Resilience Research, Mainz, Germany; 6 Department of Psychiatry and Psychotherapy, Central Institute of Mental Health, Medical Faculty Mannheim, Heidelberg University, Mannheim, Germany; 7 Hector Institute for Artificial Intelligence in Psychiatry, Central Institute of Mental Health, Medical Faculty Mannheim, Heidelberg University, Mannheim, Germany; 8 German Center for Mental Health (DZPG), partner site Mannheim-Heidelberg-Ulm, Mannheim, Germany; 9 Department of Psychiatry, Psychotherapy and Psychosomatic Medicine, Martin Luther University Halle-Wittenberg (MLU) & German Center for Mental Health (DZPG), partner site Halle-Jena-Magdeburg, Halle, Germany; 10 Department of Child and Adolescent Psychiatry, Psychosomatics and Psychotherapy, Center of Mental Health, University of Würzburg, Würzburg, Germany; 11 Division of Molecular Psychiatry, Center of Mental Health, University of Würzburg, Würzburg, Germany; 12 Department of Psychiatry and Neuropsychology, School of Mental Health and Neuroscience, Maastricht University, Maastricht, The Netherlands; 13 Fraunhofer Institute for Translational Medicine and Pharmacology ITMP, Frankfurt am Main, Germany; 14 Department of Psychiatry and Neurobehavioural Science, University College Cork, Cork, Ireland

**Keywords:** ADHD, Depression, Predictive markers, Clinical genetics

## Abstract

Attention-deficit/hyperactivity disorder (ADHD) is a highly heritable neurodevelopmental disorder, and comorbidity with other mental diseases is common. Specifically, adult ADHD (aADHD) is highly comorbid with major depressive disorder (MDD). A genetic correlation between ADHD and MDD might underlie the risk of comorbidity of both disorders. However, whether patients with ADHD and comorbid MDD differ genetically from those without comorbid MDD is currently unclear. We therefore studied the genetic background of an aADHD cohort including 352 patients with lifetime MDD and 349 patients with no history of depression, assessed by SCID-I. Polygenic risk scores for ADHD (PRS-ADHD) and MDD (PRS-MDD) were derived from large-scale genome-wide association studies. These PRS were first regressed using aADHD patients (*n* = 894) vs. healthy controls (*n* = 1026), and then using comorbidity and dimensional traits in the aADHD cohort. Both PRS-ADHD and PRS-MDD were associated with ADHD (PRS-ADHD: OR = 1.59, *p* < 0.0001; PRS-MDD: OR = 1.41, *p* < 0.0001), but only PRS-MDD was associated with comorbid MDD in aADHD patients (OR = 1.34, *p* < 0.001). Notably, patients with a history of combined MDD and anxiety disorders had the highest PRS-MDD. ADHD patients with a history of MDD had higher odds for other internalizing disorders, showed significantly more inattentive symptoms, higher neuroticism scores, lower childhood social confidence, and were more often treated as psychiatric inpatients. These findings suggest that comorbidity between aADHD and MDD is associated with genetic susceptibility to MDD, rather than neurodevelopmental factors intrinsic to ADHD pathophysiology. Our results also strengthen the view that comorbid MDD in aADHD is linked to an inattentive-internalizing rather than an impulsive-externalizing psychopathological factor.

## Introduction

Attention-deficit/hyperactivity disorder (ADHD) often presents with comorbid mental and non-mental diseases [1, 2]. Conduct and oppositional defiant disorder dominate the comorbidity pattern during childhood, whereas substance dependence, anxiety, and especially affective disorders come to the fore in adulthood [3]. Adult ADHD (aADHD) is specifically highly comorbid with major depressive disorder (MDD), and health-related well-being in aADHD negatively correlates with the severity of depressive symptoms [4]. Lifetime prevalence rates for MDD are significantly higher in aADHD patients than in the general population [5, 6]. However, a comorbid diagnosis of aADHD in MDD patients is often missed (“diagnostic over-shadowing”), particularly if patients primarily report depressive symptoms [7]. Underdiagnosis of ADHD in depressed patients may hinder treatment success, as childhood ADHD symptoms are associated with a more severe clinical manifestation of MDD during adolescence [8, 9], and patients with MDD and comorbid ADHD exhibit an overall lower quality of life and younger age at first affective episode than depressed patients without ADHD [10]. Notably, children suffering from both conditions show a particularly low social competence and academic attainment [11]. Furthermore, the genetic risk for ADHD is associated with difficult-to-treat depression (DTD) [12], and undetected ADHD is associated with worse response rates to treatment with selective serotonin reuptake inhibitors [13]. This supports previous findings that ADHD is more prevalent in DTD [14]. Some clinical features of depressive episodes, such as concentration problems, restlessness and psychomotor agitation can mimic ADHD symptoms, further complicating correct discrimination or co-diagnosis of both disorders. Therefore, the common co-occurrence of ADHD and MDD can be seen on both psychopathological and functional outcome levels. However, the etiological foundation of this connection remains unknown and is important to establish, as a better understanding of the mechanisms underlying this comorbidity might lead to earlier detection and more effective treatment of both conditions.

Currently, it is unclear how neurodevelopmental disorders and comorbid affective diseases are biologically and etiologically related to each other, especially when occurring in the same individual. One possibility is that they represent different clinical manifestations of a largely shared genetic predisposition, reflecting a common P(sychopathological)-factor which increases risk for a broad range of mental diseases [15, 16]. This would likely be reflected by an increased polygenic risk score for both ADHD (PRS-ADHD) and MDD (PRS-MDD), with high correlation between these PRS. Alternatively, it has been proposed that the polygenic risk for ADHD and neurobiological correlates (such as frontolimbic dysfunction) increase the brain’s vulnerability for depressive symptoms during later development [17, 18]. In this case, comorbidity of ADHD with MDD would be associated with a higher genetic risk for ADHD only. Lastly, it is possible that the high comorbidity of ADHD with MDD is caused by the distress of living with a neurodevelopmental disorder, and therefore a higher predisposition towards negative life events, together with an increased genetic liability to MDD. This scenario would be supported by higher genetic risk for MDD and similar genetic risk for ADHD in comorbid vs. non-comorbid patients.

Multiple genetic and environmental factors contribute to the risk of developing ADHD and, even more so, MDD. Besides psychological factors, such as demoralization by persistent adverse experiences due to internally and externally disruptive symptoms [19], peer problems, and/or poor academic attainment [20, 21], biological variables are likely to moderate the complex relationship between ADHD and co-occurring MDD. Results from twin studies have suggested a high heritability of 74% for ADHD [22] and a moderate heritability for MDD of 28–44% [23, 24]. Shared genetic factors are thought to contribute to the comorbidity of ADHD and MDD, as demonstrated by twin research [25–27], analyses of genome-wide association studies (GWAS) [28, 29], and a recent GWAS meta-analysis of ADHD and MDD [30]. However, the shared heritability between different psychiatric disorders has been derived from GWAS summary statistics to date, neglecting possible genetic overlap within a mixture of concordant and discordant genetic effects [31]. This has potentially led to an underestimation of the shared heritability. Recent research implementing a bivariate causal mixture model led to the conclusion that there are only few phenotype-specific variants and an extensive genetic overlap between disorders, with multiple pleiotropic variants with different direction and effect sizes between mental diseases, rather than disease-specific gene sets [31].

Previous studies investigating the genetic overlap between different mental diseases have typically inferred their conclusions by combining and correlating genetic risk from different GWAS for ADHD and MDD, which compare disease cases and healthy controls and do not specifically focus on the comorbidity between both conditions [28, 32]. Consequently, there is currently a lack of knowledge regarding the contribution of individual genetic predisposition to ADHD and MDD under comorbid conditions in clinical patient cohorts. This is important to determine, as the neurobiological background of ADHD with comorbid mental diseases might differ from ADHD without comorbidities. Earlier identification of aADHD patients with an increased risk for comorbid MDD may enable a targeted follow-up of such at-risk patients in longitudinal studies and stimulate preventive interventions in the future. PRS have already been shown to be useful for the identification of patients at higher risk for comorbid depression [33]. The present study investigates a clinical aADHD cohort which was thoroughly examined for depressive and other psychiatric comorbidity [34]. We characterized these patients using clinical interview data, symptom self-ratings, and genetic data with the primary aim of investigating the contribution of polygenic risk for ADHD and MDD to lifetime comorbidity with MDD in aADHD, and with the secondary aim of identifying different clinical features distinguishing aADHD patients with and without a history of/presence of MDD. Due to the high overlap between comorbid MDD and anxiety disorders (ANX) in our aADHD cohort, the high symptomatic overlap between MDD and ANX [35], and the high conversion rate of ANX into MDD [36], we additionally analyzed the effect of PRS-ADHD and PRS-MDD on comorbidity with ANX.

## Experimental procedures

### Study participants

The full patient cohort consisted of 894 adult patients (440 female, M age = 36.0, SD = 9.3; 454 male, M age = 33.2, SD = 10.8) with a diagnosis of ADHD. The cohort was recruited within the framework of a Clinical Research Unit (CRU 125). Study participants were in- and outpatients of the Department of Psychiatry, Psychosomatics and Psychotherapy, University Hospital of Würzburg, Germany. Demographic data and information about axis-I and axis-II comorbidity of the extended sample is presented elsewhere [34, 37]. Inclusion criteria were current and retrospective diagnosis of ADHD according to DSM-IV criteria. For PRS validation, a control group comprising 1026 healthy subjects (652 female, M age = 29.5, SD = 8.7; 374 male, M age = 30.6, SD = 8.3) without any history of mental disease was employed, as established by the M.I.N.I. [38]. The majority of the healthy control group (*n* = 897) was recruited within the longitudinal resilience assessment (LORA) project, described elsewhere [39]. Further healthy subjects were recruited within the framework of the CRU 125 (*n* = 129). Written informed consent was obtained from all participants, and the study was approved by the respective Ethics committees of the Universities of Würzburg (registration number 141/03), Frankfurt am Main (244/16), and Mainz (837.105.16(10424)). All studies were in accordance with the fifth revision of the Declaration of Helsinki.

For investigation of comorbidity with MDD, 73 patients had to be excluded due to missing SCID-I interview data, 117 patients were excluded due to diagnostic uncertainty regarding comorbidity with MDD due to a classification as depressive disorder other than MDD (e.g. dysthymia, mood disorders due to a general medical condition) without meeting criteria for MDD, and 3 patients were excluded due to missing documentation of ADHD diagnostic criteria, leading to a final cohort of 701 aADHD patients with both genetic and comorbidity data (Supplementary Fig. S1). In this cohort, 352 (50.2%) ADHD patients had a lifetime history of MDD, and 349 (49.8%) had no lifetime history of MDD. 86 patients (12.3%) had a current episode of MDD. 353 patients were of female sex (Age M = 35.8, SD = 9.5) and 348 of male sex (Age M = 33.3, SD = 10.7), as shown in Table 1.

**Table 1:** Demographic data, clinical characteristics and psychometric measures of the patient groups.

	MDD+	MDD−	χ²/t	OR [95% CI]/ Cohen’s D [95% CI]	*p*
**Demographic data**					
n (%)	352 (50.2)	349 (49.8)	-	-	-
Sex					
Female, n (%)	199 (56.5)	154 (44.1)	10.79	1.65 [1.22, 2.22]	**0.001**
Male, n (%)	153 (43.5)	195 (55.9)			
Age (M, SD)	35.7 (9.5)	33.4 (10.7)	3.06	0.23 [0.08, 0.38]	**0.002**
Higher secondary education, n (%)^1^	98 (28.7)	100 (30.7)	0.33	0.91 [0.65, 1.27]	0.568
Living with others, n (%)	275 (78.1)	290 (83.1)	2.77	0.73 [0.50, 1.06]	0.096
In romantic relationship n (%)^2^	216 (61.5)	221 (63.5)	0.29	0.92 [0.68, 1.25]	0.591
**Clinical characterization**					
History of psychiatric inpatient stay(s)					
Yes	99 (31.5%)	55 (17.4%)	17.19	2.19 [1.51, 3.19]	**< 0.001**
No	215 (68.5%)	262 (82.6%)			
NA	48	32			
BDI (M, SD)^3^	19.4 (10.7)	10.6 (7.6)	11.25	0.95 [0.77, 1.12]	**< 0.001**
Childhood IA symptoms (M, SD)	7.0 (1.6)	6.7 (1.8)	2.16	0.16 [0.02, 0.31]	0.031*
Childhood HI symptoms (M, SD)	5.2 (2.5)	5.5 (2.5)	1.40	0.11 [−0.04, 0.25]	0.163
Adulthood IA symptoms (M, SD)	7.5 (1.5)	7.1 (1.8)	3.36	0.25 [0.11, 0.40]	**< 0.001**
Adulthood HI symptoms (M, SD)	6.1 (1.9)	6.2 (2.0)	0.24	0.02 [−0.13, 0.17]	0.811
WURS disruptive behavior (M, SD)^4^	20.8 (9.9)	19.4 (9.9)	1.75	0.14 [−0.02, 0.30]	0.081
WURS negative affectivity (M, SD)^4^	18.3 (7.1)	14.4 (6.7)	7.00	0.56 [0.40, 0.72]	**< 0.001**
WURS social confidence (M, SD)^4^	12.8 (5.2)	14.5 (5.1)	−4.20	0.34 [0.18, 0.50]	**< 0.001**
WURS school problems (M, SD)^4^	8.6 (6.3)	8.3 (6.3)	0.51	0.04 [−0.12, 0.20]	0.613
NEO Neuroticism (M, SD)^5^	124.5 (23.1)	103.4 (25.6)	11.25	0.87 [0.71, 1.02]	**< 0.001**
**Mental health comorbidity**					
Anxiety disorders, n (%)	105 (29.8)	50 (14.3)	24.46	2.54 [1.74, 3.71]	**< 0.001**
Substance use disorders, n (%)	140 (39.8)	119 (34.1)	2.42	1.28 [0.94, 1.74]	0.120
Eating disorders, n (%)	46 (13.1)	17 (4.9)	14.40	2.94 [1.65, 5.23]	**< 0.001**
Posttraumatic stress disorders, n (%)	18 (5.1)	6 (1.7)	6.11	3.08 [1.21, 7.86]	0.013*
Somatoform disorders, n (%)	19 (5.4)	4 (1.1)	9.98	4.92 [1.66, 14.62]	**0.002**
Obsessive-compulsive disorders, n (%)	15 (4.3)	5 (1.4)	5.06	3.06 [1.10, 8.52]	0.024*

Analysis of continuous variables (age, ADHD symptoms, WURS-K, BDI) between the patient groups was performed using unpaired two-sided Student’s t-tests with effect sizes depicted by Cohen’s D values. Analysis of categorical variables was performed using Pearson’s Chi-square tests with effect sizes demonstrated by odds ratios (OR). For some of the variables, data were not available for all individuals, as indicated by ^1^ *n* = 668, ^2^ *n* = 699, ^3^ *n* = 564, ^4^ *n* = 618, ^5^ *n* = 676.

*MDD* major depressive disorder, *MDD+* indicates presence of a lifetime history of MDD; *MDD−* indicates absence of a lifetime history of MDD. *CI* confidence intervals, *M* mean, *SD* standard deviation, *n* sample sizes, *WURS* wender-utah rating scale, *BDI* beck’s depression inventory, *IA* inattentive, *HI* hyperactive/impulsive, *p* nominal p value, p* indicates p values with nominal significance at the < 0.05 level which did not survive Bonferroni correction for multiple testing. P values in bold indicate significance at the adjusted significance level ( < 0.0024).

### Clinical characterization

Demographic data and ADHD inattentive (IA) and hyperactive/impulsive (HI) symptoms (based on DSM-IV criteria) were assessed by a structured clinical interview that asked about current and childhood symptoms, retrospectively confirming onset before the age of 7. Where available, medical records, school records, and parents were consulted. In addition, self-ratings of childhood ADHD symptoms by WURS questionnaires [40] were available from 623 patients. Patients were asked whether they were ever hospitalized in a psychiatric ward. As potentially protective psychosocial variables, we obtained data regarding higher secondary education, living situation (with others vs. alone), and romantic relationship status. ADHD patients underwent the SCID-I structured clinical interview for the assessment of comorbidity with current or lifetime axis-I disorders according to DSM-IV symptom criteria [41], including MDD (Supplementary Table T1). Self-ratings of current depressive symptoms by Beck’s Depression Inventory (BDI-I) questionnaires [42] were available from 563 patients, and self-rated neuroticism scores by the NEO-PI-R instrument [43] from 673 patients. WURS factors were built based on the factor structure proposed by Calamia et al. [44] (Supplementary Table T2).

### Genotyping, quality control and imputation

Part of the cohort (*n* = 551) was genotyped using the Infinium PsychArray v1.1. Genotyping of the larger part of the overall sample (*n* = 1369) was carried out on a GSA-MD V 1.0 at the Broad Institute in Cambridge, Massachusetts, USA. Quality control of all subjects was performed using PLINK v1.9 [45]. SNPs were filtered to exclude those with minor allele frequencies ≤ 0.05, a calling rate of ≤ 0.9, variants deviating from Hardy–Weinberg equilibrium (HWE) (*p* < 1 × 10^-6^), and tri-allelic variants or variants not uniquely mappable. Participants were excluded in case of missingness > 0.1, heterozygosity rate > |0.2 |, and sex mismatch. Although all participants (case and control sample) were reported to be of Caucasian-European (CEU) origin, we assessed stratification and relatedness. Genetic principal component analysis (PCA) was performed to assess hidden population stratification. Outliers with a |SD | > 6 on one of the first 20 PCs were excluded and the sample was compared to the HapMap CEU reference sample to ensure a genetically homogeneous sample. Hence, both QC-controlled cohorts were imputed using the Michigan Imputation Server (MIS) (https://imputationserver.sph.umich.edu/), using the following parameters: phasing = eagle, rsq filter = 0.3, Population = EUR, build: hg19. Since the MIS QC did not yield any further participants missing QC criteria, we ensured a genetically homogeneous sample for our analysis. After imputation, cohorts were merged using PLINK v1.9 and underwent a second complete QC cycle as mentioned above. In addition, we carried out our analysis on a selected high-quality (HWE *p* < 0.02, MAF > 0.2, missingness = 0) SNP set that was LD pruned (r² = 0.1). In case of cryptically related subjects (pi hat > 0.2), one subject was excluded, preferentially retaining cases. After merging and second quality control, 61 cases and control participants were removed because they did not pass the QC process, leading to the final dataset of 894 aADHD patients and 1026 healthy subjects.

### Construction and validation of PRS-ADHD and PRS-MDD

PRS were calculated based on posterior effect sizes estimated using the PRS-continuous shrinkage (CS) algorithm [46]. The summary statistics used for the ADHD phenotype were from Demontis et al. [32] and for the MDD phenotype, the summary statistics were derived from Howard et al. [47]. In brief, the PRS-CS approach is based on Bayesian regression models: continuous shrinkage is applied prior to the effect sizes to include linkage disequilibrium (LD) patterns among SNPs within the datasets. PRS-CS optimizes and maximizes the number of SNPs included in PRS, since clumping and threshold methods are not required. 1000 Genomes LD patterns of the European reference panel and the default priors for effect sizes were used. Based on the posterior effect sizes estimated with PRS-CS, scores were calculated in PRSice2 [48], using the --no-clump option. While genome-wide significant loci were not reported for the ADHD GWAS within the MHC locus [32], the MDD GWAS of Howard et al. [47] reported modest and inconsistent signals in or near immune-related regions on chromosome 6, and we thus excluded the MHC region for PRS-MDD calculation in line with common practice, while keeping MHC for the construction of PRS-ADHD. The number of SNPs included in the PRS-ADHD was 882,495 and for PRS-MDD was 873,764. Only variants with an INFO score > 0.8 were considered for analysis. There is no overlap between the current sample and the samples included in the two above-mentioned GWAS summary statistics. In addition to the incremental r^2^ as an indicator of effect size, r^2^ adjusted for the liability scale (r^2^.liab) is reported based on prevalence rates of 0.03 for aADHD [49], 0.15 for MDD [50], and 0.2 for ANX [51].

### Statistical analysis

Further analyses were carried out using SPSS V29. For comparison of demographic data, clinical features, comorbidity, and psychometric measures between comorbid and non-comorbid patients (Table 1) Student’s t-tests were performed for continuous variables, and Chi-square tests for categorical variables. We report uncorrected p-values. The Bonferroni-corrected significance threshold for these analyses (21 statistical tests, Table 1) is 0.0024 for an uncorrected alpha-level of 0.05.

**Fig. 1:**
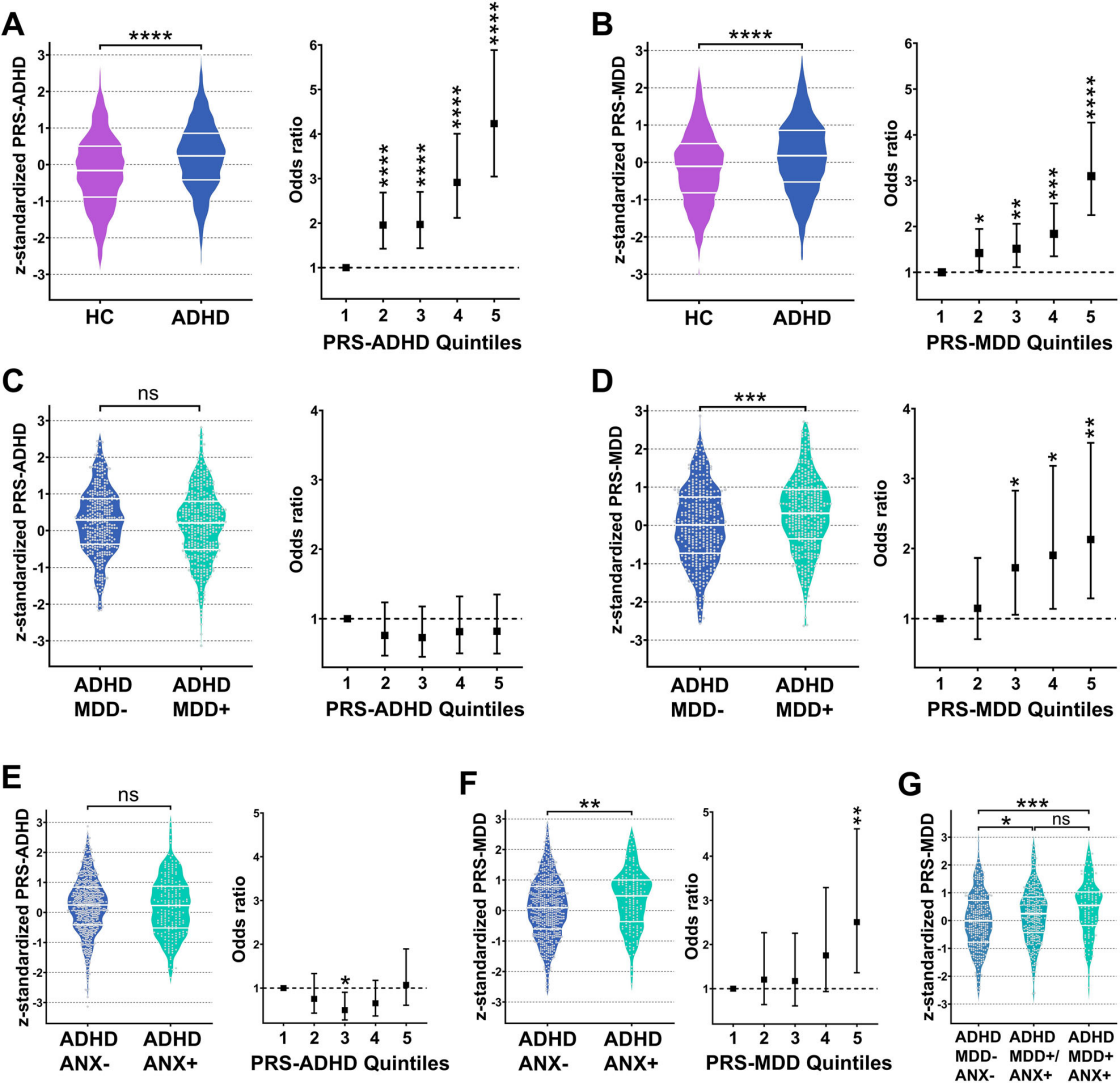
Association of ADHD and comorbid MDD with PRS-ADHD and PRS-MDD. **A** and **B** Left panels: PRS-ADHD and PRS-MDD were significantly higher in aADHD patients than in healthy controls. Right panels: Individuals with PRS in the second to fifth quintile were at higher probability for an ADHD diagnosis as compared to individuals with PRS in the lowest quintile. **C** PRS-ADHD did not differ between aADHD patients without (MDD−) and with lifetime MDD (MDD+ ), whereas **D** PRS-MDD were significantly higher in aADHD patients with a history of MDD. The same pattern showed up in aADHD patients regarding lifetime comorbidity with any anxiety disorder (ANX) which could not be discriminated by PRS-ADHD (**E**), but by PRS-MDD (**F**). **G** PRS-MDD were gradually higher in aADHD patients with either MDD or ANX (ADHD MDD+/ANX+ ) and aADHD patients with a double comorbidity with lifetime MDD and anxiety disorder (ADHD MDD+ ANX+ ) as compared to aADHD patients with neither comorbidity (ADHD MDD− ANX−). *p value < 0.05, **p value < 0.01 ***p value < 0.001, ****p value < 0.0001.

To test the main research question, we performed regression analyses with z-transformed PRS-ADHD and PRS-MDD as independent variables and non-comorbid (aADHD without lifetime MDD) vs. comorbid (aADHD with lifetime MDD) disease status as binary dependent variable, implementing age, sex, and the first five genetic PCs (for population stratification) as covariates. We additionally aimed at investigating the association of both PRS-ADHD and PRS-MDD with other axis-I disorders associated with MDD in our aADHD cohort, provided that a statistically sufficient sample size of *n* > 100 affected subjects [52] in our aADHD cohort was reached. This was only the case for ANX. Additionally, we tested PRS performance with case-control status (ADHD patients vs. healthy controls) in the full cohort. For these six binary logistic regressions (two PRS as independent variables; ADHD diagnosis, aADHD with comorbid MDD, and aADHD with comorbid ANX as dependent variables), the Bonferroni-corrected significance threshold was *p* < 0.0083. Odds ratios (OR) are reported with 95% confidence intervals (CI). For visualization of PRS distributions and between-group differences of PRS, violin plots are presented with group differences assessed by ANOVA and *post-hoc* Bonferroni-corrected t-tests. Additionally, we compared risk for the different dependent variables between the second to fifth PRS quintiles and the lowest PRS quintile by binary logistic regression analyses, again with age, sex and the first five genetic PCs as covariates (Fig. 1, Supplementary Table T3).

To estimate the incremental variance explained by the polygenic risk scores (PRS), we fitted hierarchical linear regression models for each dimensional trait (childhood and adulthood IA and HI symptoms, four different WURS factors, BDI and NEO neuroticism scores as depicted in Supplementary Table T4). The Bonferroni-corrected significance threshold for these analyses (20 statistical tests, Table 1) was 0.0025 for an uncorrected alpha-level of 0.05.

## Results

### Clinical and psychometric characterization

Statistical comparisons of the ADHD group with lifetime history of MDD and the never-depressed ADHD group with regard to demographic, clinical, and comorbidity data are depicted in Table 1.

The aADHD group with comorbid MDD consisted of significantly more women than men, while the opposite was true for the non-comorbid group (χ² = 10.79, OR = 1.65 [1.22, 2.22], *p* = 0.001). In addition, the MDD group was older than the non-comorbid patient group (t = 3.06, Cohen’s D = 0.23, *p* = 0.002). Thus, we used sex and age as demographic covariates in all following PRS analyses. Higher secondary education, living situation, and romantic relationship status did not differ between both groups (all *p* > 0.05).

Psychiatric hospitalization (ever vs. never) was significantly more prevalent in the MDD group (OR = 2.19 [1.51, 3.19], *p* = 3.38 × 10^−5^). Investigator-rated adulthood IA symptoms were higher in the MDD group (t = 3.36, Cohen’s d = 0.25, *p* = 8.29 × 10^−4^), in contrast to HI symptoms which showed no difference (*p* = 0.811 for adulthood, *p* = 0.163 for childhood). Childhood IA symptoms were nominally higher in the MDD group than in the never-depressed group (t = 2.16, *p* = 0.031), but did not pass correction for multiple testing. As expected, BDI scores were significantly higher in the lifetime MDD group than in the non-comorbid group with a large effect size (t = 11.25, Cohen’s d = 0.95, *p* = 5.02 × 10^−26^), falling within the range for clinical depression. Both the NEO neuroticism scores (Cohen’s D = 0.87, p = 4.96 × 10^−27^) and the negative affectivity factor from the WURS (Cohen’s D = 0.56, *p* = 7.32 × 10^−12^) were significantly higher in the MDD group, and childhood social confidence was rated significantly lower in the MDD group (Cohen’s D = 0.34, *p* = 3.05 × 10^−5^). Childhood school problems and disruptive behavior were not significantly different between the groups (both *p* > 0.05).

ANX (OR = 2.54 [1.74, 3.71], *p* = 7.60 × 10^−7^), eating disorders (OR = 2.94 [1.65, 5.23], p = 1.48 × 10^−4^), and somatoform disorders (OR = 4.92 [1.66, 14.62], *p* = 1.60 × 10^−3^) were more frequent in the MDD group than in aADHD patients without a lifetime history of MDD. The nominal association of MDD with posttraumatic stress disorder (OR = 3.08 [1.21, 7.86], *p* = 0.013) and obsessive-compulsive disorders (OR = 3.06 [1.10, 8.52], *p* = 0.024) did not survive correction for multiple testing. Substance abuse disorders showed no association with MDD diagnosis (*p* = 0.120).

### Association of PRS with comorbidity in ADHD

Both PRS positively correlated with each other with a weak to moderate correlation coefficient of r = 0.275 (*p* = 1.30 × 10^−34^) as shown in Supplementary Fig. S2. Both polygenic risk for ADHD and MDD were significantly associated with ADHD diagnosis (PRS-ADHD: OR = 1.59 [1.43, 1.76], *p* = 5.03 × 10^−19^, r^2^ = 0.053, r^2^.liab = 0.036; PRS-MDD: OR = 1.41 [1.26, 1.58], *p* = 6.16 × 10^−12^, r^2^ = 0.031, r^2^.liab = 0.021). Accordingly, both PRS were significantly higher in aADHD patients compared to the healthy control group (PRS-ADHD: t = 8.75, Cohen’s d = 0.40 [0.31, 0.49], *p* = 4.68 × 10^−18^; PRS-MDD: t = 6.74, Cohen’s d = 0.31 [0.22, 0.40], *p* = 2.10 × 10^−11^), with individuals in the second to fifth PRS quintiles having a higher risk for an ADHD diagnosis, in an increasing manner (Fig. 1A and B).

PRS-MDD was associated with comorbid MDD in the aADHD cohort (OR = 1.34 [1.15, 1.56], *p* = 2.33 × 10^−4^, r^2^ = 0.025, r^2^.liab = 0.024), whereas PRS-ADHD was not associated with comorbid MDD (OR = 0.90 [0.77, 1.06], *p* = 0.21), illustrated by significantly higher PRS-MDD in the aADHD MDD+ group (t = 3.64, Cohen’s D = 0.28 [0.13, 0.42], *p* = 2.99 × 10^−4^) as compared to the MDD− group, see Fig. 1C and D. In aADHD patients within the highest PRS-MDD quintile, lifetime MDD prevalence was 58% as compared to 40% in the lowest quintile (see Supplementary Fig. S3).

Due to the strong association between MDD and ANX in the investigated aADHD cohort (Table 1), the high genetic overlap between MDD and ANX [53], and the clinical relevance of comorbid ANX in adult ADHD [54], we conducted further exploratory analyses hypothesizing an association between PRS-MDD and ANX. These logistic regression analyses revealed that PRS-MDD was also associated with the broad phenotype of ANX (OR = 1.31 [1.09, 1.58], *p* = 0.004, r^2^ = 0.018, r^2^.liab = 0.023), with PRS-MDD being significantly higher in aADHD patients with a lifetime history of ANX (t = 2.92, Cohen’s D = 0.27 [0.09, 0.44], *p* = 0.004), as depicted in Fig. 1F. This association persisted even when MDD was included as additional covariate (OR = 1.25 [1.03, 1.51], *p* = 0.023). Notably, regression of PRS-MDD on a phenotype of combined MDD and ANX (*n* = 105) vs. aADHD patients without any history of MDD or ANX (n = 299) had an even better model fit with OR = 1.63 [1.27, 2.08] (*p* = 1.28 × 10^−4^, r^2^ = 0.051). A one-way ANOVA revealed that there was a significant effect of combined ANX and MDD comorbidity (independent variable: ADHD without ANX and without MDD vs. ADHD with ANX or MDD vs. ADHD with both ANX and MDD comorbidity) on PRS-MDD (F = 9.34, *p* = 9.92 × 10^−5^). *Post hoc* Bonferroni-corrected t-tests revealed significantly higher PRS-MDD in the group with one comorbidity (M = 0.22, *p* = 0.013) and in the aADHD group with double MDD and ANX comorbidity (M = 0.44, *p* = 2.20 × 10^−4^) than in the aADHD group with neither comorbidity (M = −0.02) as depicted in Fig. 1G. Again, PRS-ADHD was not associated with ANX (OR = 1.05 [0.87, 1.28], *p* = 0.60).

### Association of PRS with dimensional traits

The results of linear regression analyses depicting associations between PRS-ADHD and PRS-MDD with dimensional traits are shown in Supplementary Table T4. PRS-ADHD was nominally associated with childhood HI symptoms and childhood disruptive behavior and showed a negative association with the personality trait of neuroticism. PRS-MDD was nominally associated with childhood and adulthood HI symptoms. However, none of these associations surpassed the Bonferroni-corrected significance threshold of *p* < 0.0025 for these analyses. Other associations (e.g. PRS-ADHD with WURS negative affectivity, social confidence, and school problems; PRS-ADHD with adulthood IA and HI symptoms; or PRS-MDD with BDI scores, NEO neuroticism, and WURS negative affectivity) were also not significant.

## Discussion

We investigated the polygenic risk profile of aADHD patients with and without a history of lifetime MDD using PRS. Our data suggest that there is a distinguishable polygenic basis of both disorders when occurring together in adulthood, and that comorbid MDD in aADHD can be attributed to genetic risk factors for MDD rather than for ADHD. Furthermore, comorbid MDD was associated with other internalizing mental diseases, most significantly with ANX. Consistent with previous findings, patients with a dual comorbidity of MDD and ANX had the highest PRS-MDD [55], implying an incrementally increased risk for internalizing comorbidity with a higher polygenic load for MDD. Clinically, this internalizing profile was accompanied by substantially increased impairment: Comorbid MDD was associated with approximately twice the odds of psychiatric hospitalization. ADHD patients with a lifetime history of MDD exhibited higher inattention and a negative cognitive style, as demonstrated by higher neuroticism and childhood negative affectivity as well as lower social confidence during childhood, whereas symptoms of hyperactivity/impulsivity (HI) were not associated with depressive comorbidity. This indicates that comorbid MDD in aADHD is associated with an internalizing psychopathological factor, driven by the genetic risk for MDD.

Childhood ADHD has been consistently shown to increase the risk of subsequent MDD by longitudinal studies [56, 57]. ADHD might be associated with later depressive symptoms via mediation through ADHD-related distress, e.g. through poor academic performance, peer victimization and bullying, traumatic life events, or disturbed social relationships [11, 21, 58–61]. Such stressors might interact with genetic risk factors specific to MDD, some of which may be shared and others that are distinct between the two disorders, thereby consequently increasing the liability for comorbid MDD in ADHD. Importantly, our findings suggest that this association is not mediated by genetic liability to ADHD itself, as PRS-ADHD did not predict comorbid MDD. Rather, the comorbidity between ADHD and MDD appears to be primarily driven by genetic risk for MDD, potentially amplified by the increased risk towards negative life events associated with ADHD.

The genetic correlation between different neuropsychiatric disorders is high [28, 31] and lies between 0.40 and 0.50 for ADHD and MDD [28, 62, 63]. In our cohort, we also observed that PRS-ADHD and PRS-MDD significantly correlated with each other. Furthermore, both scores were significantly higher in the aADHD group compared to healthy controls, consistent with a general P-factor [15, 16], which increases risk for different neuropsychiatric disorders. Although a large proportion of shared heritability has been proposed between mental diseases, there is still an ongoing discussion about a disorder-specific set of causal genetic variants [31]. ADHD shares almost 80% of its causal variants with MDD and is markedly less polygenic than MDD, with an estimate of roughly 6000 causal variants for ADHD vs. 15,000 causal variants for MDD [31]. In combination with our results, this indicates that additional causal risk variants for a more heterogeneous and polygenic disorder (e.g. MDD) may be necessary to cause comorbidity with a less polygenic disorder, such as ADHD. Alternatively, increased genetic risk for ADHD interacting with environmental factors (which may be precipitated by ADHD) may be sufficient to cause comorbidity with MDD, with recent large-scale Mendelian randomization studies (MRS) supporting a causal relationship between the genetic liability for ADHD and MDD [56, 64, 65]. However, these studies draw their assumptions from different GWAS samples, which impedes an immediate comparison on the contribution of ADHD and MDD genetic risk under comorbid conditions. Also, these studies rely on the contribution of a small number of genome-wide significant SNPs [56, 65]. It has been shown that the vast majority of SNP heritability cannot be explained by genome-wide significant SNP variants, but by pleiotropic effects of variants not surpassing a genome-wide significant threshold in genetically complex neuropsychiatric diseases (“hidden heritability”). The total variance explained by genome-wide significant variants is estimated at 2–3% based on current GWAS sample sizes for most neuropsychiatric disorders [66]. Therefore, a PRS approach or a combination of MRS with a PRS approach might be more appropriate to elucidate the genetic underpinnings of mental disease comorbidity [67].

Possible differences in the genetic architecture between childhood and adulthood ADHD should also be considered when investigating the genetic underpinnings of comorbid MDD. Late-diagnosed ADHD differs from childhood ADHD (cADHD); the former displays a higher genetic overlap with depression, whereas the latter is genetically related more closely to autism spectrum disorders and hyperactivity [68]. Therefore, comorbidity patterns in aADHD and cADHD might have different genetic signatures. Alternatively, genetic variants may exert differential effects throughout neurodevelopment. MDD onset during late adolescence is mainly associated with PRS-MDD, whereas MDD onset during early adolescence is additionally associated with neurodevelopmental disorders, such as ADHD and schizophrenia [69]. Hence, the observed association in our aADHD cohort between MDD comorbidity and polygenic risk for MDD, but not polygenic risk for ADHD, may specifically reflect the situation in adult patients. It has been shown that there are two main explanatory factors for MDD: a neurodevelopmental factor and an internalizing factor [70]. In our aADHD cohort, the association of comorbid MDD with PRS-MDD, further internalizing disorders (ANX, eating disorders, somatoform disorders), neuroticism, childhood negative affectivity, and lower childhood social confidence, indicates that MDD in aADHD may be more associated with an internalizing factor than with a neurodevelopmental factor [70]. Our finding of an association between comorbid MDD and higher inattention also supports the hypothesis that comorbid MDD in aADHD is due to an internalizing psychopathological factor, as inattention and slow cognitive processing speed are both linked to internalizing problems (such as brooding, anxiety, and depression), whereas impulsivity is associated with externalizing problems (such as substance abuse, conduct disorder, and delinquency) [71–73].

The largely null associations between PRS and dimensional symptom measures are in line with evidence that PRS index broad genetic liability rather than variation of specific clinical features or quantitative traits [74, 75]. Given the modest variance explained by available ADHD PRSs [62], the restricted symptom range inherent in clinical ADHD samples, and the likelihood that self-reports reflect fluctuating symptomatic states rather than lifetime genetic liability, weak or absent associations within our aADHD cohort are not unexpected. PRS are derived from case-control GWAS. Hence, they reflect global disorder liability and combine genetic influences across heterogeneous symptom domains, limiting correlations with specific symptom dimensions such as impulsivity, hyperactivity, or negative affectivity. These considerations may explain why PRS-ADHD and PRS-MDD were associated with diagnostic status but showed no robust associations with symptom-level traits.

Our study has several strengths and limitations. The majority of PRS studies in ADHD have been conducted in childhood/adolescence cohorts, and the important matter of comorbidity with other mental diseases has not yet been addressed extensively [75]. To address this knowledge gap, we investigated the association between PRS and comorbid MDD in a clinically well-characterized single-center aADHD patient cohort with a uniform definition of related dimensional traits and comorbidities. However, due to the cross-sectional design of our study, conclusions about the causal relationship between polygenic risk scores and MDD comorbidity cannot be drawn from our data. There is also the risk of recall bias in retrospectively self-rated childhood symptoms, which might be further confounded by an excessively negative judgement in depressed individuals. In addition, we did not have data regarding age of onset of depression or detailed characteristics of the depressive disease course (e.g. number of depressive episodes). Therefore, large longitudinal studies linking the transition between childhood, adolescence, young and mid-adulthood are needed to not only capture causality between polygenic patterns and comorbidity, but to also elucidate the interaction between genetic liability, environmental risk and resilience factors, and developmental trajectories. We also did not have sufficient life event data from our cohort to analyze the interaction between polygenic risk and stressful life events. Hence, we can only hypothesize about a possible moderating role of stressful life events and other environmental risk factors for the association between genetic liability and risk of MDD comorbidity.

In conclusion, our study revealed that comorbid MDD in aADHD was associated with polygenic risk for MDD, increased symptoms of inattention, and with a negative cognitive style, as indicated by higher neuroticism, higher childhood negative affectivity, and lower childhood social confidence. Our results therefore suggest that aADHD comorbidity with MDD may be due to an inattentive-internalizing factor rather than a neurodevelopmental psychopathological factor, and that comorbid MDD in aADHD has its own unique genetic contribution, despite significant overlap with the polygenic risk for ADHD. Hence, the significant and distressing burden of living with ADHD with possible impairment in different domains of daily life alone might not be sufficient to explain comorbidity with ANX and MDD in ADHD. A distinguishable polygenic pattern for comorbidity with internalizing mental disorders in patients with neurodevelopmental disorders, such as ADHD, should be considered as a plausible alternative concept parallel to the broad genetic P-factor. Future longitudinal studies should aim to clarify how biological, environmental and psychopathological signatures may be causally related.

## Supplementary information

Supplementary Material

## Data Availability

The data that support the findings of this study are available from the corresponding author upon reasonable request.

## References

[1] Kittel-Schneider S, Arteaga-Henriquez G, Vasquez AA, Asherson P, Banaschewski T, Brikell I, et al. Non-mental diseases associated with ADHD across the lifespan: fidgety philipp and pippi longstocking at risk of multimorbidity? Neurosci Biobehav Rev. 2021. 10.1016/j.neubiorev.2021.10.035 34757108

[2] McGough JJ, Smalley SL, McCracken JT, Yang M, Del’Homme M, Lynn DE, et al. Psychiatric comorbidity in adult attention deficit hyperactivity disorder: findings from multiplex families. Am J Psychiatry. 2005;162:1621–7. 16135620 10.1176/appi.ajp.162.9.1621

[3] Franke B, Michelini G, Asherson P, Banaschewski T, Bilbow A, Buitelaar JK, et al. Live fast, die young? a review on the developmental trajectories of ADHD across the lifespan. Eur Neuropsychopharmacol. 2018;28:1059–88. 30195575 10.1016/j.euroneuro.2018.08.001 PMC6379245

[4] Ahnemark E, Di Schiena M, Fredman AC, Medin E, Soderling JK, Ginsberg Y. Health-related quality of life and burden of illness in adults with newly diagnosed attention-deficit/hyperactivity disorder in Sweden. BMC Psychiatry. 2018;18:223. 30005675 10.1186/s12888-018-1803-y PMC6044069

[5] Choi WS, Woo YS, Wang SM, Lim HK, Bahk WM. The prevalence of psychiatric comorbidities in adult ADHD compared with non-ADHD populations: a systematic literature review. PLoS One. 2022;17:e0277175. 36331985 10.1371/journal.pone.0277175 PMC9635752

[6] Hartman CA, Larsson H, Vos M, Bellato A, Libutzki B, Solberg BS, et al. Anxiety, mood, and substance use disorders in adult men and women with and without attention-deficit/hyperactivity disorder: a substantive and methodological overview. Neurosci Biobehav Rev. 2023;151:105209. 37149075 10.1016/j.neubiorev.2023.105209

[7] McIntosh D, Kutcher S, Binder C, Levitt A, Fallu A, Rosenbluth M. Adult ADHD and comorbid depression: a consensus-derived diagnostic algorithm for ADHD. Neuropsychiatr Dis Treat. 2009;5:137–50. 19557108 10.2147/ndt.s4720 PMC2695217

[8] Powell V, Agha SS, Jones RB, Eyre O, Stephens A, Weavers B, et al. ADHD in adults with recurrent depression. J Affect Disord. 2021;295:1153–60. 34706428 10.1016/j.jad.2021.09.010 PMC8552915

[9] Meinzer MC, Pettit JW, Waxmonsky JG, Gnagy E, Molina BS, Pelham WE. Does childhood attention-deficit/hyperactivity disorder (ADHD) predict levels of depressive symptoms during emerging adulthood?. J Abnorm Child Psychol. 2016;44:787–97. 26272531 10.1007/s10802-015-0065-0 PMC4754165

[10] McIntyre RS, Kennedy SH, Soczynska JK, Nguyen HT, Bilkey TS, Woldeyohannes HO, et al. Attention-deficit/hyperactivity disorder in adults with bipolar disorder or major depressive disorder: results from the international mood disorders collaborative project. Prim Care Companion J Clin Psychiatry. 2010;12:PCC.09m00861. 20944770 10.4088/PCC.09m00861gry PMC2947541

[11] Blackman GL, Ostrander R, Herman KC. Children with ADHD and depression: a multisource, multimethod assessment of clinical, social, and academic functioning. J Atten Disord. 2005;8:195–207. 16110050 10.1177/1087054705278777

[12] Fabbri C, Hagenaars SP, John C, Williams AT, Shrine N, Moles L, et al. Genetic and clinical characteristics of treatment-resistant depression using primary care records in two UK cohorts. Mol Psychiatry. 2021;26:3363–73. 33753889 10.1038/s41380-021-01062-9 PMC8505242

[13] Sternat T, Fotinos K, Fine A, Epstein I, Katzman MA. Low hedonic tone and attention-deficit hyperactivity disorder: risk factors for treatment resistance in depressed adults. Neuropsychiatr Dis Treat. 2018;14:2379–87. 30271154 10.2147/NDT.S170645 PMC6149933

[14] Bron TI, Bijlenga D, Verduijn J, Penninx BW, Beekman AT, Kooij JJ. Prevalence of ADHD symptoms across clinical stages of major depressive disorder. J Affect Disord. 2016;197:29–35. 26970265 10.1016/j.jad.2016.02.053

[15] Sprooten E, Franke B, Greven CU. The P-factor and its genomic and neural equivalents: an integrated perspective. Mol Psychiatry. 2021;27:38–48. 33526822 10.1038/s41380-021-01031-2 PMC8960404

[16] Caspi A, Houts RM, Belsky DW, Goldman-Mellor SJ, Harrington H, Israel S, et al. The p factor: one general psychopathology factor in the structure of psychiatric disorders?. Clin Psychol Sci. 2014;2:119–37. 25360393 10.1177/2167702613497473 PMC4209412

[17] Biederman J, Newcorn J, Sprich S. Comorbidity of attention deficit hyperactivity disorder with conduct, depressive, anxiety, and other disorders. Am J Psychiatry. 1991;148:564–77. 2018156 10.1176/ajp.148.5.564

[18] Kebets V, Favre P, Houenou J, Polosan M, Perroud N, Aubry JM, et al. Fronto-limbic neural variability as a transdiagnostic correlate of emotion dysregulation. Transl Psychiatry. 2021;11:545. 34675186 10.1038/s41398-021-01666-3 PMC8530999

[19] Biederman J, Mick E, Faraone SV. Depression in attention deficit hyperactivity disorder (ADHD) children: “true” depression or demoralization?. J Affect Disord. 1998;47:113–22. 9476751 10.1016/s0165-0327(97)00127-4

[20] Humphreys KL, Katz SJ, Lee SS, Hammen C, Brennan PA, Najman JM. The association of ADHD and depression: mediation by peer problems and parent-child difficulties in two complementary samples. J Abnorm Psychol. 2013;122:854–67. 24016021 10.1037/a0033895 PMC3806877

[21] Powell V, Riglin L, Hammerton G, Eyre O, Martin J, Anney R, et al. What explains the link between childhood ADHD and adolescent depression? investigating the role of peer relationships and academic attainment. Eur Child Adolesc Psychiatry. 2020;29:1581–91. 31932968 10.1007/s00787-019-01463-w PMC7595988

[22] Faraone SV, Larsson H. Genetics of attention deficit hyperactivity disorder. Mol Psychiatry. 2019;24:562–75. 29892054 10.1038/s41380-018-0070-0 PMC6477889

[23] Kendler KS, Gatz M, Gardner CO, Pedersen NL. A Swedish national twin study of lifetime major depression. Am J Psychiatry. 2006;163:109–14. 16390897 10.1176/appi.ajp.163.1.109

[24] Fernandez-Pujals AM, Adams MJ, Thomson P, McKechanie AG, Blackwood DH, Smith BH, et al. Epidemiology and heritability of major depressive disorder, stratified by age of onset, sex, and illness course in generation scotland: scottish family health study (GS:SFHS). PLoS One. 2015;10:e0142197. 26571028 10.1371/journal.pone.0142197 PMC4646689

[25] Rydell M, Taylor MJ, Larsson H. Genetic and environmental contributions to the association between ADHD and affective problems in early childhood-A Swedish population-based twin study. Am J Med Genet B Neuropsychiatr Genet. 2017;174:538–46. 28436115 10.1002/ajmg.b.32536

[26] Segenreich D, Paez MS, Regalla MA, Fortes D, Faraone SV, Sergeant J, et al. Multilevel analysis of ADHD, anxiety and depression symptoms aggregation in families. Eur Child Adolesc Psychiatry. 2015;24:525–36. 25156273 10.1007/s00787-014-0604-1

[27] Chen TJ, Ji CY, Wang SS, Lichtenstein P, Larsson H, Chang Z. Genetic and environmental influences on the relationship between ADHD symptoms and internalizing problems: a Chinese twin study. Am J Med Genet B Neuropsychiatr Genet. 2016;171:931–7. 26710920 10.1002/ajmg.b.32411

[28] Anttila V, Bulik-Sullivan B, Finucane HK, Walters RK, Bras J, Duncan L, et al. Analysis of shared heritability in common disorders of the brain. Science. 2018;360:eaap8757. 29930110 10.1126/science.aap8757 PMC6097237

[29] Du Rietz E, Coleman J, Glanville K, Choi SW, O’Reilly PF, Kuntsi J. Association of polygenic risk for attention-deficit/hyperactivity disorder with co-occurring traits and disorders. Biol Psychiatry Cogn Neurosci Neuroimaging. 2018;3:635–43. 30047479 10.1016/j.bpsc.2017.11.013 PMC6278881

[30] Powell V, Martin J, Thapar A, Rice F, Anney RJL. Investigating regions of shared genetic variation in attention deficit/hyperactivity disorder and major depressive disorder: a GWAS meta-analysis. Sci Rep. 2021;11:7353. 33795730 10.1038/s41598-021-86802-1 PMC8016853

[31] Hindley G, Frei O, Shadrin AA, Cheng W, O’Connell KS, Icick R, et al. Charting the landscape of genetic overlap between mental disorders and related traits beyond genetic correlation. Am J Psychiatry. 2022;179:833–43. 36069018 10.1176/appi.ajp.21101051 PMC9633354

[32] Demontis D, Walters GB, Athanasiadis G, Walters R, Therrien K, Nielsen TT, et al. Genome-wide analyses of ADHD identify 27 risk loci, refine the genetic architecture and implicate several cognitive domains. Nat Genet. 2023;55:198–208. 36702997 10.1038/s41588-022-01285-8 PMC10914347

[33] Cao Z, Yang H, Ye Y, Zhang Y, Li S, Zhao H, et al. Polygenic risk score, healthy lifestyles, and risk of incident depression. Transl Psychiatry. 2021;11:189. 33782378 10.1038/s41398-021-01306-w PMC8007584

[34] Gross-Lesch S, Dempfle A, Reichert S, Jans T, Geissler J, Kittel-Schneider S, et al. Sex- and Subtype-Related Differences in the Comorbidity of Adult ADHDs. J Atten Disord. 2016;20:855–66. 24196345 10.1177/1087054713510353

[35] Nutt D. Anxiety and depression: individual entities or two sides of the same coin?. Int J Psychiatry Clin Pract. 2004;8:19–24. 24930685 10.1080/13651500410005513

[36] Batterham PJ, Christensen H, Calear AL. Anxiety symptoms as precursors of major depression and suicidal ideation. Depress Anxiety. 2013;30:908–16. 23494924 10.1002/da.22066

[37] Jacob CP, Gross-Lesch S, Reichert S, Geissler J, Jans T, Kittel-Schneider S, et al. Sex- and subtype-related differences of personality disorders (Axis II) and personality traits in persistent ADHD. J Atten Disord. 2016;20:1056–65. 24510476 10.1177/1087054714521293

[38] Sheehan DV, Lecrubier Y, Sheehan KH, Amorim P, Janavs J, Weiller E, et al. The mini-international neuropsychiatric interview (M.I.N.I.): the development and validation of a structured diagnostic psychiatric interview for DSM-IV and ICD-10. J Clin Psychiatry. 1998;59:22–33. 9881538

[39] Chmitorz A, Neumann RJ, Kollmann B, Ahrens KF, Ohlschlager S, Goldbach N, et al. Longitudinal determination of resilience in humans to identify mechanisms of resilience to modern-life stressors: the longitudinal resilience assessment (LORA) study. Eur Arch Psychiatry Clin Neurosci. 2021;271:1035–51. 32683526 10.1007/s00406-020-01159-2 PMC8354914

[40] Ward MF, Wender PH, Reimherr FW. The Wender Utah Rating Scale: an aid in the retrospective diagnosis of childhood attention deficit hyperactivity disorder. Am J Psychiatry. 1993;150:885–90. 8494063 10.1176/ajp.150.6.885

[41] First MB, Spitzer RL, Gibbon M, Williams JBW Structured Clinical Interview for DSM-IV Axis I Disorders, Clinician Version (SCID-CV). Washington, DC: American Psychiatric Press; 1996.

[42] Richter P, Werner J, Heerlein A, Kraus A, Sauer H. On the validity of the beck depression inventory. A review Psychopathology. 1998;31:160–8. 9636945 10.1159/000066239

[43] Costa PT, McCrae RR Revised NEO Personality Inventory (NEO-PI-R) and NEO Five Factor Inventory: Professional manual. Odessa, FL: Psychological Assessment Resources. 1992.

[44] Calamia M, Hill BD, Musso MW, Pella RD, Gouvier WD. Factor structure and clinical correlates of the 61-item Wender Utah Rating Scale (WURS). Atten Defic Hyperact Disord. 2018;10:177–88. 29427262 10.1007/s12402-018-0251-3

[45] Chang CC, Chow CC, Tellier LC, Vattikuti S, Purcell SM, Lee JJ. Second-generation PLINK: rising to the challenge of larger and richer datasets. Gigascience. 2015;4:7. 25722852 10.1186/s13742-015-0047-8 PMC4342193

[46] Ge T, Chen CY, Ni Y, Feng YA, Smoller JW. Polygenic prediction via bayesian regression and continuous shrinkage priors. Nat Commun. 2019;10:1776. 30992449 10.1038/s41467-019-09718-5 PMC6467998

[47] Howard DM, Adams MJ, Clarke TK, Hafferty JD, Gibson J, Shirali M, et al. Genome-wide meta-analysis of depression identifies 102 independent variants and highlights the importance of the prefrontal brain regions. Nat Neurosci. 2019;22:343–52. 30718901 10.1038/s41593-018-0326-7 PMC6522363

[48] Choi SW, O’Reilly PF. PRSice-2: polygenic risk score software for biobank-scale data. Gigascience. 2019;8:giz082. 31307061 10.1093/gigascience/giz082 PMC6629542

[49] Ayano G, Tsegay L, Gizachew Y, Necho M, Yohannes K, Abraha M, et al. Prevalence of attention deficit hyperactivity disorder in adults: umbrella review of evidence generated across the globe. Psychiatry Res. 2023;328:115449. 37708807 10.1016/j.psychres.2023.115449

[50] Wray NR, Ripke S, Mattheisen M, Trzaskowski M, Byrne EM, Abdellaoui A, et al. Genome-wide association analyses identify 44 risk variants and refine the genetic architecture of major depression. Nat Genet. 2018;50:668–81. 29700475 10.1038/s41588-018-0090-3 PMC5934326

[51] Baxter AJ, Scott KM, Vos T, Whiteford HA. Global prevalence of anxiety disorders: a systematic review and meta-regression. Psychol Med. 2013;43:897–910. 22781489 10.1017/S003329171200147X

[52] Choi SW, Mak TS, O’Reilly PF. Tutorial: a guide to performing polygenic risk score analyses. Nat Protoc. 2020;15:2759–72. 32709988 10.1038/s41596-020-0353-1 PMC7612115

[53] Penninx BW, Pine DS, Holmes EA, Reif A. Anxiety disorders. Lancet. 2021;397:914–27. 33581801 10.1016/S0140-6736(21)00359-7 PMC9248771

[54] Quenneville AF, Kalogeropoulou E, Nicastro R, Weibel S, Chanut F, Perroud N. Anxiety disorders in adult ADHD: a frequent comorbidity and a risk factor for externalizing problems. Psychiatry Res. 2022;310:114423. 35152068 10.1016/j.psychres.2022.114423

[55] Coombes BJ, Landi I, Choi KW, Singh K, Fennessy B, Jenkins GD, et al. The genetic contribution to the comorbidity of depression and anxiety: a multi-site electronic health records study of almost 178 000 people. Psychol Med. 2023;53:7368–74. 38078748 10.1017/S0033291723000983 PMC10719682

[56] Riglin L, Leppert B, Dardani C, Thapar AK, Rice F, O’Donovan MC, et al. ADHD and depression: investigating a causal explanation. Psychol Med. 2021;51:1890–7. 32249726 10.1017/S0033291720000665 PMC8381237

[57] Gundel LK, Pedersen CB, Munk-Olsen T, Dalsgaard S. Longitudinal association between mental disorders in childhood and subsequent depression - a nationwide prospective cohort study. J Affect Disord. 2018;227:56–64. 29053976 10.1016/j.jad.2017.10.023

[58] Sahmurova A, Arikan S, Gursesli MC, Duradoni M. ADHD symptoms as a stressor leading to depressive symptoms among university students: the mediating role of perceived stress between ADHD and depression. Int J Env Res Public Health. 2022;19:11091. 36078805 10.3390/ijerph191711091 PMC9518099

[59] Becker SP, Mehari KR, Langberg JM, Evans SW. Rates of peer victimization in young adolescents with ADHD and associations with internalizing symptoms and self-esteem. Eur Child Adolesc Psychiatry. 2017;26:201–14. 27315106 10.1007/s00787-016-0881-y PMC6048591

[60] Roy A, Hartman CA, Veenstra R, Oldehinkel AJ. Peer dislike and victimisation in pathways from ADHD symptoms to depression. Eur Child Adolesc Psychiatry. 2015;24:887–95. 25348085 10.1007/s00787-014-0633-9

[61] Powell V, Riglin L, Ng-Knight T, Frederickson N, Woolf K, McManus C, et al. Investigating friendship difficulties in the pathway from adhd to depressive symptoms. can parent-child relationships compensate?. Res Child Adolesc Psychopathol. 2021;49:1031–41. 33655375 10.1007/s10802-021-00798-w PMC8222013

[62] Demontis D, Walters RK, Martin J, Mattheisen M, Als TD, Agerbo E, et al. Discovery of the first genome-wide significant risk loci for attention deficit/hyperactivity disorder. Nat Genet. 2019;51:63–75. 30478444 10.1038/s41588-018-0269-7 PMC6481311

[63] Du Rietz E, Pettersson E, Brikell I, Ghirardi L, Chen Q, Hartman C, et al. Overlap between attention-deficit hyperactivity disorder and neurodevelopmental, externalising and internalising disorders: separating unique from general psychopathology effects. Br J Psychiatry. 2021;218:35–42. 32892757 10.1192/bjp.2020.152

[64] Soler Artigas M, Sanchez-Mora C, Rovira P, Vilar-Ribo L, Ramos-Quiroga JA, Ribases M. Mendelian randomization analysis for attention deficit/hyperactivity disorder: studying a broad range of exposures and outcomes. Int J Epidemiol. 2023;52:386–402. 35690959 10.1093/ije/dyac128 PMC10114062

[65] Garcia-Argibay M, Brikell I, Thapar A, Lichtenstein P, Lundstrom S, Demontis D, et al. Attention-deficit/hyperactivity disorder and major depressive disorder: evidence from multiple genetically informed designs. Biol Psychiatry. 2023. 10.1016/j.biopsych.2023.07.017 37562520

[66] O’Connell KS, Coombes BJ. Genetic contributions to bipolar disorder: current status and future directions. Psychol Med. 2021;51:2156–67. 33879273 10.1017/S0033291721001252 PMC8477227

[67] Shen X, Howard DM, Adams MJ, Hill WD, Clarke TK, Major Depressive Disorder Working Group of the Psychiatric Genomics C, et al. A phenome-wide association and Mendelian Randomisation study of polygenic risk for depression in UK Biobank. Nat Commun. 2020;11:2301. 32385265 10.1038/s41467-020-16022-0 PMC7210889

[68] Rajagopal VM, Duan J, Vilar-Ribo L, Grove J, Zayats T, Ramos-Quiroga JA, et al. Differences in the genetic architecture of common and rare variants in childhood, persistent and late-diagnosed attention-deficit hyperactivity disorder. Nat Genet. 2022;54:1117–24. 35927488 10.1038/s41588-022-01143-7 PMC10028590

[69] Rice F, Riglin L, Thapar AK, Heron J, Anney R, O’Donovan MC, et al. Characterizing developmental trajectories and the role of neuropsychiatric genetic risk variants in early-onset depression. JAMA Psychiatry. 2019;76:306–13. 30326013 10.1001/jamapsychiatry.2018.3338 PMC6439821

[70] Grotzinger AD, Mallard TT, Akingbuwa WA, Ip HF, Adams MJ, Lewis CM, et al. Genetic architecture of 11 major psychiatric disorders at biobehavioral, functional genomic and molecular genetic levels of analysis. Nat Genet. 2022;54:548–59. 35513722 10.1038/s41588-022-01057-4 PMC9117465

[71] Sevincok D, Ozbay HC, Ozbek MM, Tunagur MT, Aksu H. ADHD symptoms in relation to internalizing and externalizing symptoms in children: the mediating role of sluggish cognitive tempo. Nord J Psychiatry. 2020;74:265–72. 31809238 10.1080/08039488.2019.1697746

[72] So FK, Chavira D, Lee SS. ADHD and ODD dimensions: time varying prediction of internalizing problems from childhood to adolescence. J Atten Disord. 2022;26:932–41. 34632828 10.1177/10870547211050947

[73] Takeda T, Ambrosini PJ, deBerardinis R, Elia J. What can ADHD without comorbidity teach us about comorbidity?. Res Dev Disabil. 2012;33:419–25. 22119689 10.1016/j.ridd.2011.09.024

[74] Jangmo A, Brikell I, Kuja-Halkola R, Feldman I, Lundstrom S, Almqvist C, et al. The association between polygenic scores for attention-deficit/hyperactivity disorder and school performance: The role of attention-deficit/hyperactivity disorder symptoms, polygenic scores for educational attainment, and shared familial factors. JCPP Adv. 2021;1:e12030. 37431440 10.1002/jcv2.12030 PMC10242908

[75] Ronald A, de Bode N, Polderman TJC. Systematic review: how the Attention-Deficit/Hyperactivity disorder polygenic risk score adds to our understanding of ADHD and associated traits. J Am Acad Child Adolesc Psychiatry. 2021;60:1234–77. 33548493 10.1016/j.jaac.2021.01.019 PMC11164195

