# Supplementary material for: Adult ADHD with comorbid major depression shows a distinguishable polygenic pattern and negative cognitive style
Source: Transl Psychiatry. 2026 Apr 4;16:235. doi: 10.1038/s41398-026-04008-3 (PMC13079749; doi:10.1038/s41398-026-04008-3)
Supplement: Supplementary file 1 — Supplementary Material [file 41398_2026_4008_MOESM1_ESM.docx]

- Supplementary Information -

**Adult ADHD with comorbid major depression shows a distinguishable polygenic pattern and negative cognitive style**

Thorsten M. Kranz, Rhiannon V. McNeill, Christian P. Jacob, Kira F. Ahrens, Rebecca J. Neumann, Michael M. Plichta, Bianca Kollmann, Fabian Streit, Oliver Tüscher, Klaus Lieb, Heike Weber, Marcel Romanos, Klaus-Peter Lesch, Andreas Reif, Sarah Kittel-Schneider, Georg C. Ziegler


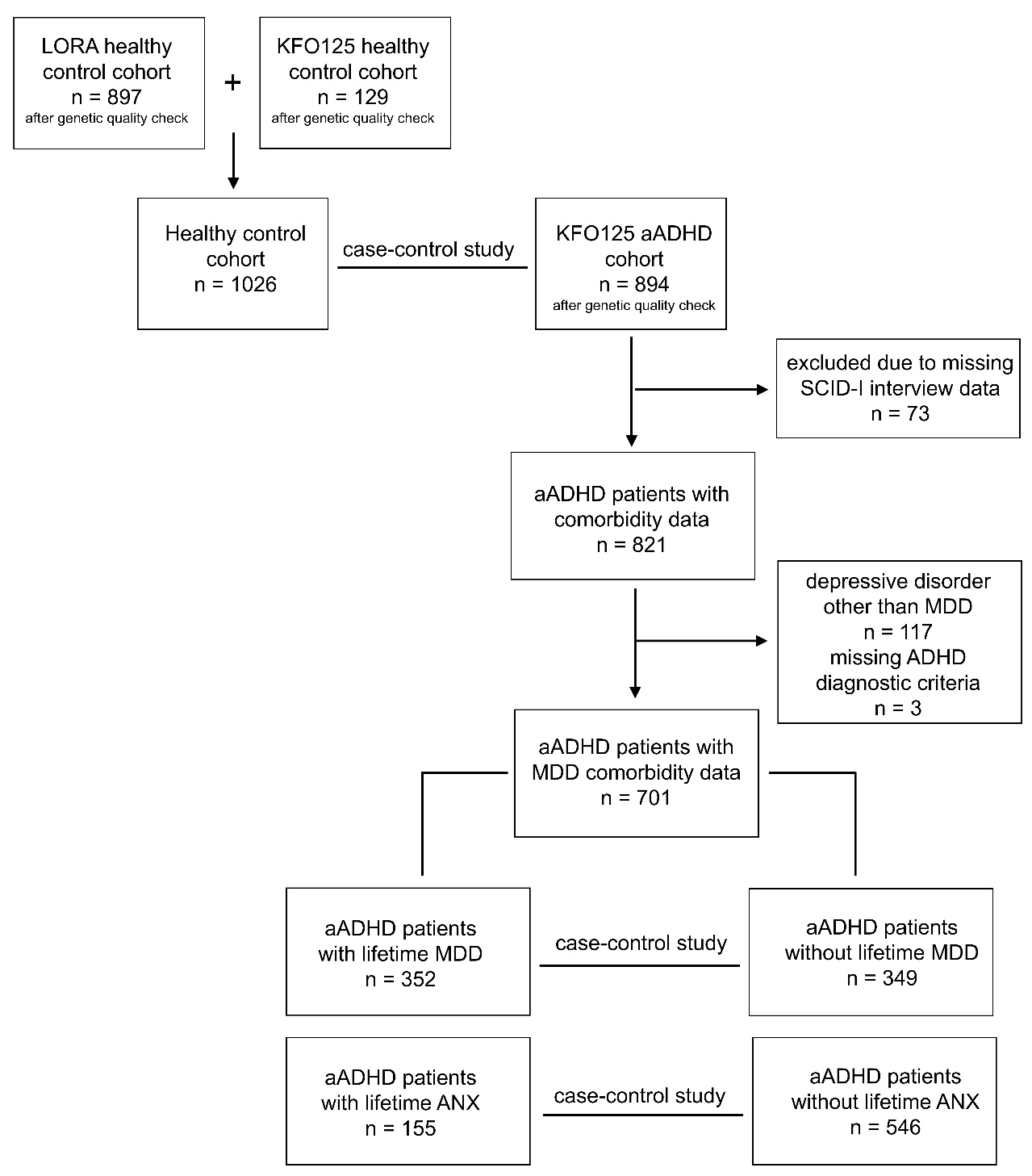


**Fig. S1** Flowchart showing the cohorts under study and case-control studies performed


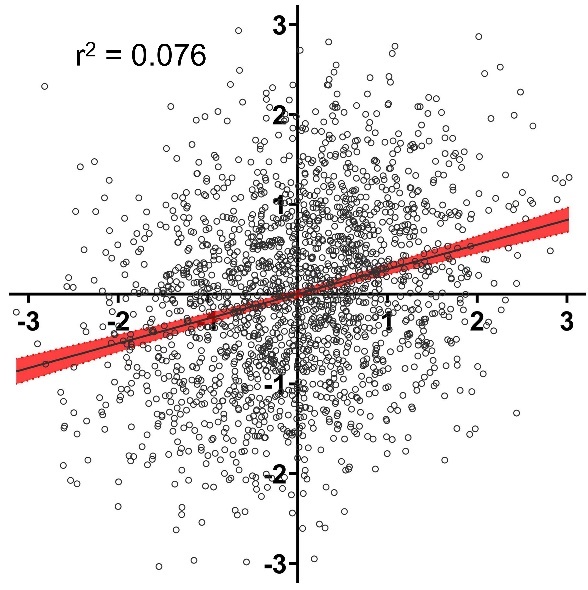


**Fig. S2** Correlation between PRS-ADHD and PRS-MDD

The Graph shows the correlation between PRS-ADHD (x-axis) and PRS-MDD (y-axis) in the full cohort (adult ADHD and healthy controls). PRS are z-standardized.

r: Pearson correlation coefficient.


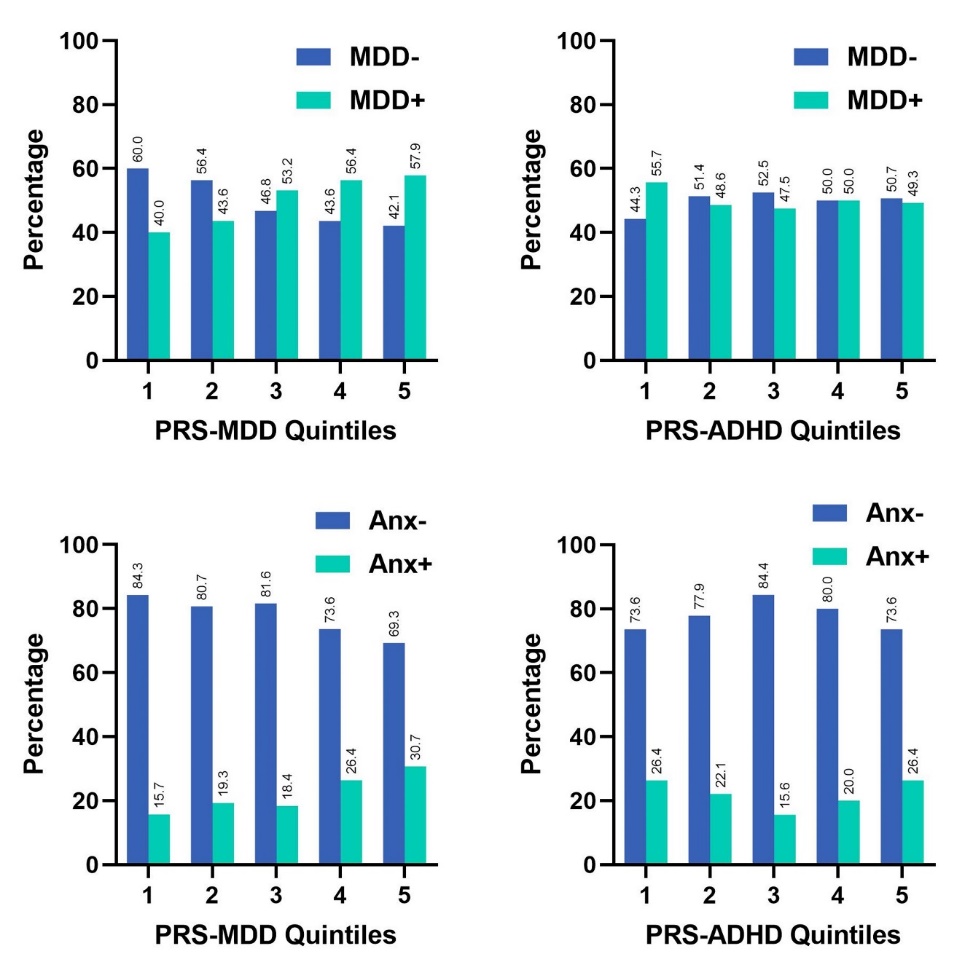


**Fig. S3** Prevalence of lifetime comorbidities stratified by PRS quintiles

Graphs show percentages of lifetime prevalence with MDD (MDD+/MDD-, upper panel) and anxiety disorders (Anx-/Anx+, lower panel) stratified by quintiles of PRS-MDD and PRS-ADHD. For PRS-MDD quintiles there is a stepwise increase in comorbidity rates both for MDD and ANX, whereas there is no such effect for PRS-ADHD. MDD: Major depressive disorder, Anx: Anxiety disorders, n = 140 for quintiles 1, 2, 4, and 5 and n = 141 for quintile 3.

**Table T1** Construction of diagnostic categories

| **Diagnostic category** | **Diagnoses** |
| --- | --- |
| Major depressive disorder (MDD) | Narrow category of MDD (without dysthymia, mood disorders due to a general medical condition) |
| Anxiety disorders | Panic disorder  Agoraphobia  Social phobia  Generalized anxiety disorder  Anxiety disorder due to a general medical condition |
| Substance use disorders | Abuse and/or  Dependency of relevant substance groups |
| Eating disorders | Anorexia nervosa  Bulimia nervosa  Binge eating disorder |
| Posttraumatic stress disorders | Posttraumatic stress disorders |
| Somatoform disorders | Somatization disorder  Somatoform disorder  Pain disorder |
| Obsessive-compulsive disorders | Obsessive-compulsive disorder  Hypochondria  Body dysmorphic disorder |

**Table T2** WURS items organized according to the factor structure proposed by Calamia et al. (2018)

| **Disruptive behavior** | **Neuroticism** | **Social confidence** | **School problems** |
| --- | --- | --- | --- |
| 7. Hot- or short-tempered,  low boiling point | 2. Afraid of things | 1. Active, restless, always  on the go | 51. Overall a poor student,  slow learner |
| 9. Temper outbursts,  tantrums | 4. Anxious, worrying | 8. Shy, sensitive (-) | 52. Slow in *learning* to read |
| 11. Stubborn, strong-willed | 5. Nervous, fidgety | 18. Outgoing, friendly,  enjoyed company of  people | 53. Slow reader |
| 13. Incautious. dare-devilish,  involved in pranks | 12. Sad or blue, depressed,  unhappy | 22. Friends, popular | 54. Trouble reversing letters |
| 15. Disobedient with parents,  rebellious, sassy | 14. Not getting a kick out of  things, dissatisfied with  life | 29. Unpopular with other  children, didn’t keep  friends for long, didn’t get  along with other children  (-) | 55. Problems with spelling |
| 21. Angry | 16. Low opinion of myself | 32. Well-coordinated, picked  first in games | 56. Trouble with  mathematics/numbers |
| 27. Losing control of myself | 17. Irritable | 37. Leader, bossy | 60. Repeating grades |
| 28. Tendency to be or act  irrational | 20. Moody, ups and downs |  |  |
| 34. Running away from home | 26. Guilty feelings, regretful |  |  |
| 35. Getting into fights |  |  |  |
| 36. Teasing other children |  |  |  |
| 40. Trouble seeing things  from someone else’s  point of view |  |  |  |
| 41. Trouble with authorities,  trouble with school, visits  to principal’s office |  |  |  |
| 42. Trouble with police,  booked, convicted |  |  |  |
| 61. Suspended or expelled |  |  |  |

| **IV: PRS-ADHD quintiles, DV: ADHD diagnosis** | | | |
| --- | --- | --- | --- |
| **Quintile** | **OR** | **95% CI** | **n** |
| 1 | 1 | 1.00, 1.00 | 384 |
| 2 | 1.96**** | 1.43, 2.69 | 384 |
| 3 | 1.97**** | 1.44, 2.70 | 384 |
| 4 | 2.91**** | 2.12, 4.01 | 384 |
| 5 | 4.23**** | 3.05, 5.89 | 384 |
| **IV: PRS-MDD quintiles, DV: ADHD diagnosis** | | | |
| **Quintile** | **OR** | **95% CI** | **n** |
| 1 | 1.00 | 1.00, 1.00 | 384 |
| 2 | 1.42* | 1.04, 1.95 | 384 |
| 3 | 1.51** | 1.11, 2.06 | 384 |
| 4 | 1.84*** | 1.35, 2.50 | 384 |
| 5 | 3.10**** | 2.25, 4.27 | 384 |
| **IV: PRS-ADHD quintiles, DV: ADHD + MDD** | | | |
| **Quintile** | **OR** | **95% CI** | **n** |
| 1 | 1.00 | 1.00, 1.00 | 140 |
| 2 | 0.76 | 0.47, 1.24 | 140 |
| 3 | 0.73 | 0.45, 1.18 | 141 |
| 4 | 0.81 | 0.50, 1.32 | 140 |
| 5 | 0.82 | 0.50, 1.35 | 140 |
| **IV: PRS-MDD quintiles, DV: ADHD + MDD** | | | |
| **Quintile** | **OR** | **95% CI** | **n** |
| 1 | 1.00 | 1.00, 1.00 | 140 |
| 2 | 1.15 | 0.71, 1.87 | 140 |
| 3 | 1.73* | 1.06, 2.83 | 141 |
| 4 | 1.90* | 1.14, 3.18 | 140 |
| 5 | 2.13** | 1.29, 3.51 | 140 |
| **IV: PRS-ADHD quintiles, DV: ADHD + ANX** | | | |
| **Quintile** | **OR** | **95% CI** | **n** |
| 1 | 1.00 | 1.00, 1.00 | 140 |
| 2 | 0.75 | 0.43, 1.33 | 140 |
| 3 | 0.50* | 0.27, 0.90 | 141 |
| 4 | 0.65 | 0.36, 1.18 | 140 |
| 5 | 1.07 | 0.61, 1.89 | 140 |
| **IV: PRS-MDD quintiles, DV: ADHD + ANX** | | | |
| **Quintile** | **OR** | **95% CI** | **n** |
| 1 | 1.00 | 1.00, 1.00 | 140 |
| 2 | 1.21 | 0.64, 2.27 | 140 |
| 3 | 1.17 | 0.61, 2.26 | 141 |
| 4 | 1.75 | 0.93, 3.29 | 140 |
| 5 | 2.51** | 1.36, 4.62 | 140 |

**Table T3** Logistic regression results for PRS quintiles

IV: independent variable, DV: dependent variable. Age, sex, and the first five principal components were set as covariates. OR: odds ratios for respective PRS quintiles vs. the first quintile as reference, CI: confidence interval, n: sample size. * p value < .05, ** p value < .01 *** p value < .001, **** p value < .0001.

|  | **Beta (SE)** | **95% CI** | Δ **pseudo r^2^** | ***p*** |
| --- | --- | --- | --- | --- |
| **PRS-ADHD (z-standardized)** | | | | |
| BDI | 0.027 (0.411) | -0.780, 0.834 | 0.000 | .947 |
| Childhood IA symptoms | 0.037 (0.063) | -0.086, 0.161 | 0.000 | .556 |
| Childhood HI symptoms | 0.267 (0.092) | 0.087, 0.448 | 0.010 | .004* |
| Adulthood IA symptoms | -0.051 (0.058) | -0.165, 0.062 | 0.001 | .376 |
| Adulthood HI symptoms | 0.099 (0.073) | -0.043, 0.242 | 0.002 | .173 |
| NEO neuroticism | -2.112 (0.93) | -3.947, -0.277 | 0.006 | .024* |
| WURS negative affectivity | 0.154 (0.302) | -0.440, 0.748 | 0.000 | .610 |
| WURS disruptive behavior | 1.093 (0.413) | 0.282, 1.904 | 0.011 | .008* |
| WURS social confidence | 0.393 (0.221) | -0.041, 0.827 | 0.005 | .076 |
| WURS school problems | 0.424 (0.268) | -0.103, 0.952 | 0.004 | .114 |
| **PRS-MDD (z-standardized)** | | | | |
| BDI | 0.48 (0.386) | -0.269, 1.245 | 0.002 | .206 |
| Childhood IA symptoms | -0.001 (0.06) | -0.116, 0.119 | 0.000 | .982 |
| Childhood HI symptoms | 0.239 (0.087) | 0.068, 0.411 | 0.009 | .006* |
| Adulthood IA symptoms | 0.017 (0.055) | -0.091, 0.125 | 0.000 | .754 |
| Adulthood HI symptoms | 0.116 (0.069) | -0.031, 0.302 | 0.007 | .016* |
| NEO neuroticism | 1.405 (0.890) | -0.342, 3.153 | 0.003 | .115 |
| WURS negative affectvity | 0.219 (0285) | -0.340, 0.779 | 0.001 | .442 |
| WURS disruptive behavior | 0.585 (0.391) | -0.183, 1.352 | 0.003 | .134 |
| WURS social confidence | 0.225 (0.209) | -0.185, 0.635 | 0.002 | .281 |
| WURS school problems | -0.343 (0.253) | -0.841 0.154 | 0.003 | .175 |

**Table T4** Association of PRS-ADHD and PRS-MDD with dimensional traits

Results of hierarchical linear regression models with age, sex, and the first five genetic principal components entered in the first block, followed by z-standardized PRS in the second block. Beta: unstandardized regression coefficient, CI: confidence interval, Δ pseudo r^2^: Incremental variance explained by PRS. IA: inattentive, HI: hyperactive/impulsive, WURS: Wender-Utah Rating Scale, p: uncorrected p value, p* indicates nominally significant p values at an uncorrected significance threshold of < .05 not surpassing the Bonferroni-corrected significance threshold of p < .0025.

**References**

1. Calamia, M., Hill, B. D., Musso, M. W., Pella, R. D., & Gouvier, W. D. (2018). Factor structure and clinical correlates of the 61-item Wender Utah Rating Scale (WURS). *Atten Defic Hyperact Disord, 10*(3), 177-188. doi:10.1007/s12402-018-0251-3
